# Coat colour in dogs: identification of the *Merle *locus in the Australian shepherd breed

**DOI:** 10.1186/1746-6148-2-9

**Published:** 2006-02-27

**Authors:** Benoit Hédan, Sébastien Corre, Christophe Hitte, Stéphane Dréano, Thierry Vilboux, Thomas Derrien, Bernard Denis, Francis Galibert, Marie-Dominique Galibert, Catherine André

**Affiliations:** 1 UMR 6061 CNRS, Génétique et Développement, Faculté de Médecine, Université de Rennes1, 35043 RENNES Cédex, France.; 2 5 avenue Foch 54200 Toul, France.

## Abstract

**Background:**

Coat colours in canines have many natural phenotypic variants. Some of the genes and alleles involved also cause genetic developmental defects, which are also observed in humans and mice. We studied the genetic bases of the *merle *phenotype in dogs to shed light on the pigmentation mechanisms and to identify genes involved in these complex pathways. The *merle *phenotype includes a lack of eumelanic pigmentation and developmental defects, hearing impairments and microphthalmia. It is similar to that observed in *microphthalmia *mouse mutants.

**Results:**

Taking advantage of the dog as a powerful genetic model and using recently available genomic resources, we investigated the segregation of the *merle *phenotype in a five-generation pedigree, comprising 96 sampled Australian shepherd dogs. Genetic linkage analysis allowed us to identify a locus for the *merle *phenotype, spanning 5.5 megabases, at the centromeric tip of canine chromosome 10 (CFA10). This locus was supported by a Lod score of 15.65 at a recombination fraction θ = 0. Linkage analysis in three other breeds revealed that the same region is linked to the *merle *phenotype. This region, which is orthologous to human chromosome 12 (HSA12 q13-q14), belongs to a conserved ordered segment in the human and mouse genome and comprises several genes potentially involved in pigmentation and development.

**Conclusion:**

This study has identified the locus for the *merle *coat colour in dogs to be at the centromeric end of CFA10. Genetic studies on other breeds segregating the *merle *phenotype should allow the locus to be defined more accurately with the aim of identifying the gene. This work shows the power of the canine system to search for the genetic bases of mammalian pigmentation and developmental pathways.

## Background

Coat colours in mammals depend on skin and hair pigment synthesis. Melanocytes manufacture two types of melanin: the black/brown photo-protective eumelanin pigment, and the red-yellow cytotoxic phaeomelanin pigment. Several paracrine factors secreted primarily by surrounding keratinocytes are involved in the melanogenic pathway by stimulating the switch between phaeomelanin and eumelanin [1]. In this pathway, *microphthalmia transcription factor *(*MITF*) plays a central role by regulating the expression of the *TYR *(*Tyrosinase), TRP-1 *(*Tyrosine Related Protein*) and *DCT *(*Dopachrome Tautomerase*) genes that encode enzymes involved in pigment manufacture [2,3].

Coat colour is highly polymorphic in dogs. In 1957, Little described, after observing the possible phenotypes, more than 20 loci affecting coat colours [4,5]. Until recently, only a few genes were recognised as involved in pigmentation. However, more and more genes, alleles and new interactions are being discovered: variants of *melanocortine 1 receptor *gene (*MC1R*), (locus previously called *extension *E) [6-8], variants of Agouti, the antagonist ligand of MC1R [9,10], variants of *tyrosinase-related protein 1 *(*TYRP1*) [11] and variants of *melanophillin *[12]. Three mutations responsible for the brown coat colour versus black coat colour were described in *TYRP1 *in several dog breeds including the Australian Shepherd dog [11]. Genomic tools are now fully available in canine genetics: dense radiation hybrid maps with 1500 polymorphic microsatellite markers and anchored BAC markers [13,14], a radiation hybrid map comprising 10,000 canine gene-based markers [15], and a whole sequence assembly of the canine genome, build 2.1 [16]. Altogether, the dog appears to be a good model for understanding better the genetics of pigmentation in mammals and for isolating new genes, new variants and interactions between alleles of different loci.

**Figure 1:**
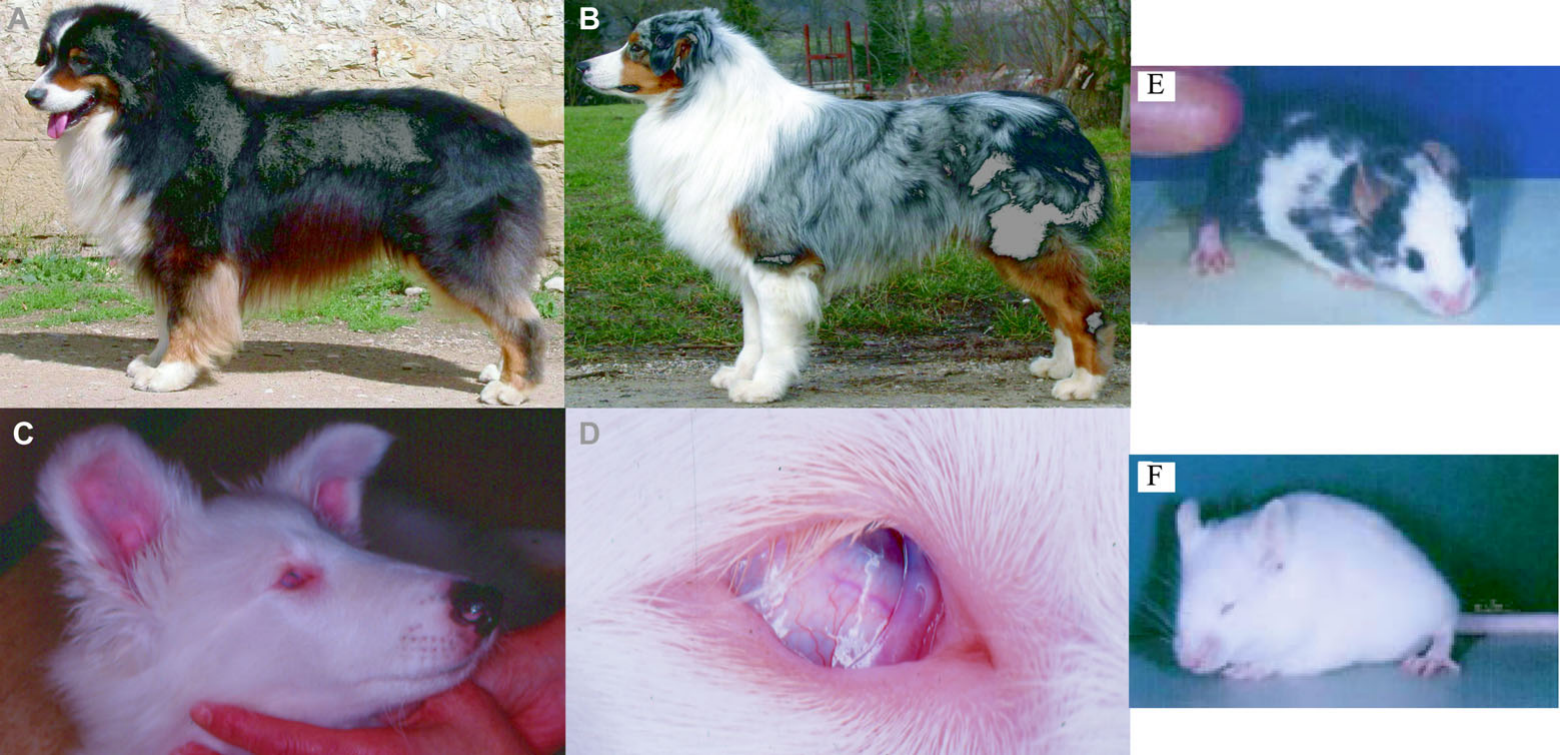
**Pictures of none *merle *and *merle *dogs and mice microphthalmia mutants**. A: Black and tan Australian Shepherd dog. B: Heterozygous *merle *Australian shepherd dog (pictures from Elevage du Paradis Sauvage de Ménestruel, Poncin, France) [40]. C: Six-month homozygous *merle *collie: the coat colour is totally white and the dog is blind and deaf. D: The left eye of the same dog, shows a microphthalmia with microcornea and a cataract (pictures from Dr Gilles Chaudieu, DVM, Dip. ECVO, Chamalières, France). E: *Mitf*^*mi*-*sp*^/*Mitf*^*mi*-*vga9 *^mouse. F: *Mitf*^*mi*-*vga9*^/*Mitf*^*mi*-*vga9 *^mouse with severe microphthalmia (pictures from Steingrímsson [23]).

We are interested in the *merle *phenotype because of its involvement in coat colour and developmental impairments. The *merle *phenotype is a dominant trait, with heterozygous dogs presenting a coat colour in which eumelanic regions are incompletely and irregularly diluted, leaving intensely pigmented patches. *Merle *is found throughout the body except on the pheomelanic regions of the black and tan coat colour (Figure 1A, 1B). These dogs often have heterochromia iridis or blue eyes and often have a lack of retinal pigment visible on the fundus. Homozygous *merle *dogs display a more severe phenotype. The dogs are usually very pale, sometimes completely white and present developmental defects with an incomplete penetrance, microphthalmia and hearing loss (Figure 1C, 1D). In *merle *European lineages, microphthalmia and/or hearing loss are not frequently observed as breeders avoid mating *merle *dogs to avoid these developmental defects. However, several veterinary studies on the "*merle *syndrome", reported retinal defects [17], microphthalmia and coloboma [18]. The non-survival or degeneration of melanocytes in the cochlea have been suggested to explain hearing loss [19].

When analysing the genetic basis of the *merle *phenotype, Little suggested that a unique locus (called M) was responsible for the *merle *phenotype in different breeds [4]. It was proposed that the *merle *coat colour may be due to a transposable element, after the observation of two germinal reversions out of 66 *merle *offspring of a homozygous *merle *female [20]. Recently, the *Kit Ligand*, *KITLG*, was excluded as a candidate gene for the *merle *phenotype in dogs [21] and the candidate gene approach has not yet give any conclusive results.

We searched for candidate genes for the *merle *phenotype in dogs by considering well-described pigment disorders in mice. Mutations in the gene of the Mitf pathway cause specific coat colour phenotypes, some of which are similar to the *merle *phenotype in dogs. These include dilution of the coat colour in patches and complete or mild microphthalmia (Figure 1E, 1F). Also, the complete abolition of functional Mitf results in loss of the melanocyte lineage, causing a white coat colour due to a lack of pigment cell manufacturer, and additional eye (microphthalmia) and inner ear disorders. Heteroallelic combinations of *MITF *variants produce animals with normal sized dark eyes and yellowish-brown to grey spotted checker board-like coat colours [22,23]. Mitf is also involved in human Waardenburg syndromes, including pigment cell migration disorders [24] and developmental defects such as deafness. Pax3 (Paired box gene 3) and Sox10 (SRY – Sex determining region Y-box 10), which regulates *MITF *gene expression, are also associated with this syndrome [25,26]. This genetic evidence suggest that *MITF, PAX3 *and *SOX10 *genes may be candidate genes for the *merle *phenotype.

We collected a pedigree of Australian shepherd dogs and used a genetic linkage approach with microsatellite markers flanking the *MITF, PAX3 *and *SOX10 *candidate genes to search for the genetic bases of the *merle *coat colour in canines. Although these three genes were excluded for the *merle *phenotype in dogs, we successfully identified the *merle *locus on canine chromosome 10, close to the centromere, 20 Mb away from Sox10. This locus was restricted to a 5.5 Mb interval and was further confirmed by analysing families of other breeds segregating the *merle *phenotype.

## Results

### Pedigrees

**Figure 2:**
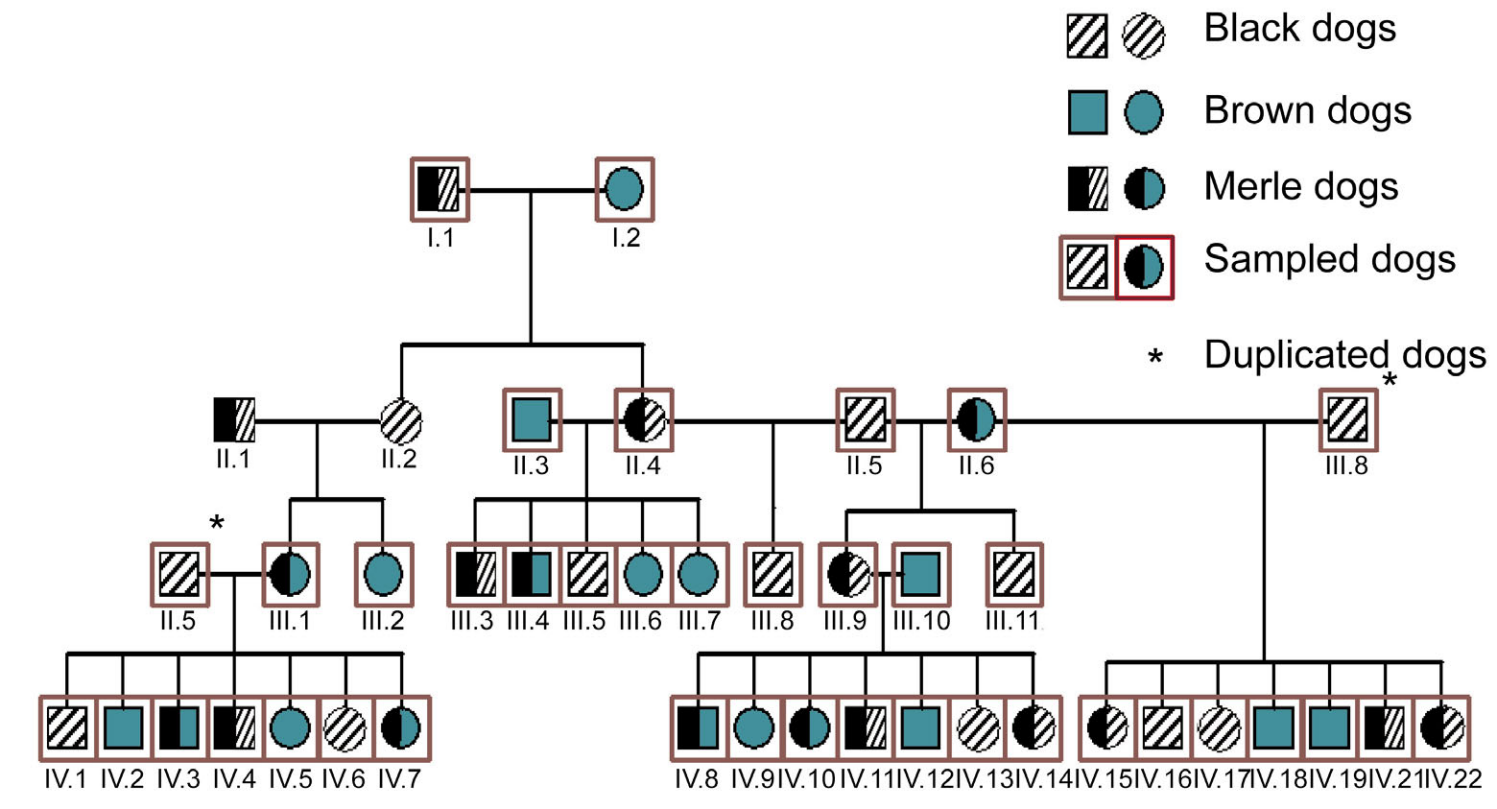
Sub-pedigree of 38 sampled Australian shepherd dogs (19 brown and 19 black dogs).

A pedigree comprising 96 Australian shepherd dogs (43 brown and 53 black dogs) was collected. This pedigree, called the "complete pedigree", included 42 *merle *dogs. A sub-pedigree of 38 dogs, including 17 *merle *dogs, derived from the complete pedigree was used for genotyping (Figure 2). Isolated families from different breeds segregating the *merle *coat colour were also collected, including three dachshund families (14 dogs); a Beauce shepherd family (five dogs) and a Border collie family (13 dogs).

### Genetic linkage analysis of the brown coat colour in the pedigrees

We evaluated the linkage power of the pedigrees by investigating the genetic linkage between the brown phenotype and the *TYRP1 *gene in the complete pedigree. As the *TYRP1 *gene was previously associated with the brown coat colour in dogs [11], we genotyped markers linked to *TYRP1*. These markers, FH2319 and REN105I03, are 1.18 and 5.17 Mb from *TYRP1*, respectively (see Additional file 1). The Lod scores between the brown phenotype and FH2319 and REN105I03 markers obtained by a two-point analysis on the complete pedigree, were 7.2 and 3.5 respectively, with a recombination fraction of θ = 0. For the sub-pedigree, the Lod scores were 3.6 and 2.4 respectively at θ = 0. The number of informative meiosis for the brown phenotype was 58 in the complete pedigree and 16 in the sub-pedigree. As the number of informative meiosis for the *merle *phenotype significantly increased to 81 and 33 in the complete pedigree and sub-pedigree, respectively, we expected these present pedigrees to be valuable for linkage analysis of the *merle *phenotype.

### Genetic linkage analysis of the *merle *trait

As expected, the transmission mode in the collected pedigrees was consistent with an autosomal dominant segregation of the *merle *phenotype.

Using the sub-pedigree, we carried out genetic linkage analysis on polymorphic markers either flanking or within the intronic part of *TYRP1 *gene and the candidate genes *MITF*, *PAX3*, and *SOX 10*. Two-point analysis showed no significant linkage between the *merle *phenotype and markers flanking the *MITF *and *PAX3 *genes (Table 1). Therefore, we could exclude *MITF *and *PAX3 *being involved in this phenotype. However, we found significant linkages (Lod scores ranging from 3.09 to 3.65) with markers flanking *SOX10*, with recombination fractions, θ, ranging from 0.08 to 0.14 (Table 2). This suggested that the *merle *locus was about 10 cM from *SOX10*. As part of a "chromosome walking strategy", we selected 30 new polymorphic markers spanning a 27 Mb region from the *SOX10 *region to the centromeric tip of CFA10, as the C10.769 marker telomeric to *SOX10 *had a decreasing Lod score (Table 2).

Linkage analysis allowed us to identify seven markers close to the centromere, CFA10.1 to CFA10.8, which cosegregate with the *merle *phenotype with significant Lod scores (> 3) (Table 2, see Additional file 2).

**Figure 3:**
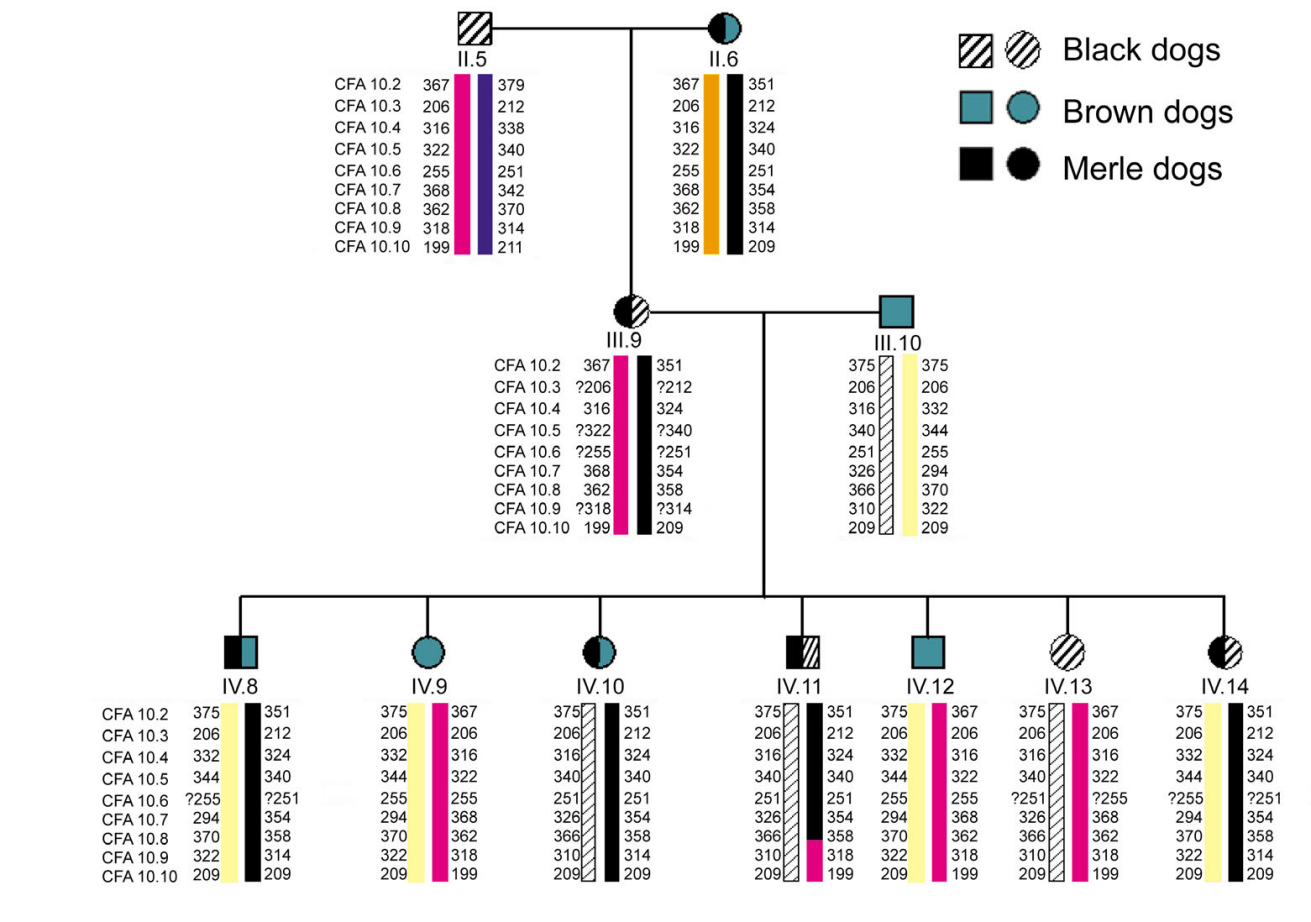
**Haplotype analysis of the centromeric end of CFA10**. Analysis revealed a crossing-over between CFA10.8 and CFA10.9 markers (dog IV.11), limiting the *merle *locus in between the centromeric end and the CFA10.9 marker. The *merle *chromosome is shown in black. Alleles with a question mark indicate that the parental origin of the allele could not be determined.

We extended the genetic linkage by analysing the nine most centromeric markers in the "complete pedigree" and in three nuclear families of dachshund, Beauce shepherd and Border collie segregating the *merle *phenotype. We obtained increased Lod scores for markers CFA10.1 to CFA10.8, with maximum Lod scores for CFA10.7 and CFA10.8 (Lod scores at θ = 0 of 15.65 and 14.90 in the complete pedigree and 19.87 and 19.57 in the complete pedigree plus the three other families, respectively). The CFA10.9 marker (telomeric to CFA10.8) is unlinked to the phenotype (Lod scores at θ = 0 of -14.38 in the complete pedigree and -11.97 in the complete pedigree plus the three other families, respectively, Table 2). Haplotype analyses of this region allowed us to detect a recombination event between the *merle *phenotype and the CFA10.9 microsatellite, thus limiting further the *merle *locus (Figure 3). These data, as well as the previous results for *SOX10 *flanking markers (Table 2), allowed us to exclude the *SOX10 *gene as being involved in the *merle *phenotype. Our results show that the *merle *locus is located in a 5.5 Mb region between the end of the centromere, arbitrarily located at 3 Mb, (represented by CFA10.1 located at 3.1 Mb) and the CFA10.9 marker (located at 8.5 Mb) defining the telomeric limit of the critical interval (see Additional file 2).

## Discussion

In the present study, we used a genetic linkage approach on a pedigree of Australian shepherd dogs segregating the *merle *phenotype. We identified with high statistical support a 5.5 Mb locus at the centromeric tip of CFA10 in which the gene responsible for this phenotype should be found. Dog samples were collected from breeders. As phenotyping was easily and immediately detectable after birth by breeders themselves and then officially declared to the breed club, we have been able to obtain an informative family with as many as five generations of Australian shepherd dogs. Moreover, parentage testing ensured good reliability of the pedigrees. Such collected pedigrees from existing families means that housing of dogs is not required, limiting housing costs and ethical issues.

We focused on three candidate genes belonging to the coat colour pathway: *MITF, PAX3 *and *SOX 10*. The *merle *phenotype shares similarities with *Mitf *mouse mutants in coat colour and ocular and hearing defects, and also with human Waardenburg patients. However, our genetic linkage study ruled out these three genes as being involved in the *merle *phenotype. Genetic analysis of the *SOX10 *region in the sub-pedigree, using a "chromosome walking" strategy on CFA10, allowed us to identify the *merle *locus. It spans a 5.5 Mb region 20 Mb away from *SOX10*. Genetic linkage analyses on the complete pedigree and on small families from other breeds confirmed that the *merle *locus was located between the CFA10 centromere (3 Mb) and the CFA10.9 marker (8.5 Mb), with the highest Lod score of 19.87.

The corresponding orthologous human region is HSA12q13-q14 (position from 54.36 Mb to 60.94 Mb) and mouse region is MMU10D3 (position from 122.8 Mb to 128.7 Mb). These orthologous dog, human and mouse regions correspond to a unique conserved ordered segment, which has the same orientation in dogs and humans but is inverted between dogs and mice. In the dog region, 99 genes are predicted and 48 are known (Broad1), in the human region, 134 genes are predicted and 98 are known (NCBI 35), and in the mouse region, 112 genes are predicted and 95 are known (NCBI M34) [27]. These gene numbers may vary due to slight changes in the annotated genes as the versions are updated. In humans, mice and dogs, the conserved segments are totally ordered, making annotation of the dog segment easy, thus helping determination of candidate *merle *genes. This locus has many candidate genes, with at least a dozen being potential metabolic candidates as they, or their paralogs, belong to the pigmentation pathway. These include proteins involved in neural crest development (such as ERBB3), melanosome motility and transfer to surrounding keratinocytes (such as Silv/Pmel-17 and rab, kinesin, dynactin, myosin proteins).

Although the *MITF *gene itself has been excluded, the *merle *mutation should affect a gene interacting directly with the *MITF *gene in the pigmentation pathway. Alternatively, a more complex mechanism could explain the incomplete penetrance of eye defects observed in homozygous *merle *dogs. Although hearing loss may be due to an extreme white phenotype, including the absence of melanocytes in the cochlea, as in other white canine breeds [28], less is known about the origins of microphthalmia and other ocular defects. These may be due to another mutation in the same locus.

The *merle *phenotype occurs in several breeds and is commonly encountered in mongrel dogs. Breeds segregating *merle *are from the collie lineage (Group 1-FCI – Federation Cynologique Internationale classification): Shetland sheepdog, Border collie, collie, Australian shepherd dog, etc., and from other unrelated breeds belonging to different FCI groups and different clusters, as defined by Parker *et al*. [29], such as dachshund (Group 4-FCI):, Beauce shepherd (Group 1-FCI), great Dane (Group 2-FCI), Welsh corgi cardigan and Pyrenean shepherd. The *merle *phenotype is most probably very old, with the *merle *coat colour being reported in old books [30,31], from which drawings of *merle *dogs have been selected and reproduced [32].

It is not yet known whether the genetic cause of the *merle *phenotype is the same in all breeds and mongrels segregating this phenotype. A unique locus has been suggested as responsible for the *merle *coat colour [4]. In the present study, the increased Lod scores observed for genotyped markers from the *merle *locus in dachshund, Beauce shepherd and Border collie families is consistent, at least in these breeds, with there being a unique locus for the *merle *coat colour. If all *merle *dogs share a common ancestor chromosome, all breeds segregating *merle *could be used to refine the locus. The sharing of the *merle *locus by several breeds and also by mongrels may be due either to a common ancestor chromosome region being transmitted throughout canine evolution and/or to backcrosses that introduced a *merle *haplotype in several breeds at different times.

## Conclusion

Using genetic linkage analysis, we excluded the involvement of the *MITF, PAX3 *and *SOX 10 *candidates genes in the *merle *phenotype. However, we identified the *merle *locus at the centromeric end of CFA10 in pedigrees of Australian shepherd dogs, dachshund, Beauce shepherd dogs and Border collies segregating the *merle *phenotype. This locus spans 5.5 Mb and is linked to the *merle *coat colour with a maximum Lod score of 19.87 and a recombination fraction of 0. We are currently analysing this locus in several breeds segregating *merle*, with a high density of single nucleotide polymorphic markers (SNP). This should help in identifying the *merle *gene. As well as benefiting breeding practices and canine veterinary medicine, identifying the *merle *gene will also help in understanding the genetic bases of mammalian pigmentation and developmental pathways.

## Methods

### Genomic DNA extraction

No dogs were housed for research purposes, and all dogs were privately owned pets.

Blood samples and the accompanying pedigree and coat colour data (with pictures when possible) were collected by DVM veterinarians. All data were entered into a database. Genomic DNA was extracted from 5 ml of blood collected on EDTA, using the nucleon BACC 3 kit (Amersham Biosciences, Piscataway, NJ, USA). For low concentration samples, the extracted DNA was "whole genome amplified" using the genomiphi kit (Amersham Biosciences).

### Canine pedigree

Pedigrees were constructed using the Cyrillic software (Cyrillic2.1) [33], which allows haplotypes from the genotyping data to be drawn and the data to be exported in different formats for use in genetic linkage analysis. We carried out genotyping of 20 polymorphic microsatellites from four different chromosomes (CFA10, 11, 20 and 37) to check and validate the parentage compatibility.

### Markers selection and Genotyping experiments

Microsatellite markers were selected from RH map data [13,34] or from the CanFam 1.0 draft of the canine genome sequence [35]. Markers were selected from their position and their polymorphism level (see Additional file 1). We used Primer3 software to design PCR primers [36].

Microsatellite markers were labelled using a two-step-PCR fluorescent labelling procedure [37]. The first step was carried out on 50 ng of dog genomic DNA using a classical PCR protocol and a touchdown program of 61°C to 51°C. The second step consisted of a one strand labelling PCR as previously described [37]. The PCR products were purified using Sephadex G50 fine column filtration in a 96 format (Amersham Biosciences). An aliquot of 3 μl fluorescent purified PCR product was mixed with 0.3 μl (0.2 nM) of fluorescent geneScan-500 ROX size standard (Applied Biosystems) and 8.7 μl of formamide, and then loaded onto a 3130 XL genetic analyser (Applied Biosystems). Results were analysed using GeneMapper software v3.7 (Applied Biosystems) and the genotyping data were used by Cyrillic software for the genetic linkage analysis.

### Genetic linkage analysis

Haplotypes were constructed using the Cyrillic software. Two-point linkage analysis was carried out between each marker and the *merle *phenotype using M-LINK software through the GLUE web interface [38] and MultiMap software [39]. We used the 'prepare' option of CRI-MAP to check for Mendelian segregation. The linkage between each pair of markers was carried out with the TWOPOINT option of CRI-MAP. Lod scores were calculated assuming an autosomal dominant transmission with full penetrance and affected individuals were scored as heterozygous at the phenotype locus.

## Authors' contributions

BH collected samples, constructed the pedigrees and performed genotyping experiments; BH also interpreted all dataand actively participated in writing the manuscript. SC helped with the genotyping experiments and interpretation of the dataand participated in writing the manuscript. CH carried out the statistical analyses and data interpretation, and critically revised the manuscript. SD helped with the genotyping experiments. TV extracted DNA from blood samples and commented critically on the work and manuscript. TD carried out the synteny analyses. BD contributed with knowledge on canines coat colours and critically revised the manuscript. FG provided intellectual input and critically revised the manuscript. MDG helped conceive and design the work and helped in the writing of the manuscript. CA conceived and designed the work and drafted the manuscript. All authors read and approved the final manuscript.

## Supplementary Material

Additional File 1 **Characteristics of the markers used in the genetic linkage studies**. ^a ^CFA : *Canis familiaris *chromosome.^b ^Starred markers were selected from the CanFam 1.0 canine sequence draft, ^c ^marker corresponding to marker FH2537 from Guyon et al. [13], ^d ^number of alleles as determined from the sub-pedigree. ^E ^Markers flanking *SOX10 *gene. Click here for file

Additional File 2 **Scheme of two-point linkage analysis of the *merle *phenotype in the Australian Shepherd dog pedigrees on CFA10**. Two-point linkage analysis of the *merle *phenotype in the Australian shepherd dog sub-pedigree (in black) and complete pedigree (in brown) (Lod scores at theta = 0) is shown on the right. An ordered list of genotyped markers (right) and genes (left) and their position in Mb are indicated in the middle. An ideogram of the canine chromosome 10 is shown on the left with the corresponding human chromosomal conserved segments. NB: genomic sequence systematically starts at an arbitrary coordinate of 3 Mb to include the non-sequenced centromeric region. Click here for file

## Figures and Tables

**Table 1:** Linkage data between the *merle *phenotype and markers flanking the *TYRP1 *gene and *MITF *and *PAX3 *candidate genes. Two point Lod scores and recombination fractions (θ) between the *merle *phenotype and the markers flanking *TYRP1*, *MITF *and *PAX3 *genes calculated on the sub-pedigree. ^a ^CFA: *Canis familiaris *chromosome. ^b ^Starred markers were selected from the CanFam 1.0 canine sequence draft. Significant exclusions (<-2) are indicated in bold.

			θ at Lod score max		Lod scores at θ						
CFA^a^	*Gene*/Markers^b^	Gene/Marker Position in bp	θ	Lod score max	0.001	0.01	0.05	0.1	0.15	0.2	0.3

**CFA11**	***TYRP1***	34,664779–34,683148									
	FH2319	35,863893–35,864169	0.5	0.0	**-23.083**	**-13.146**	**-6.442**	**-3.802**	**-2.429**	-1.582	-0.661
	Ren105l03	39,857761–39,857999	0.5	0.0	**-15.888**	**-8.939**	**-4.278**	**-2.471**	-1.549	-0.995	-0.417

	Ren96j16	22,981531–22,981669	0.5	0.0	**-13.194**	**-8.198**	**-4.721**	**-3.24**	**-2.384**	-1.786	-0.963
	Ren130E03	22,981511–22,981683	0.5	0.0	**-13.194**	**-8.198**	**-4.721**	**-3.24**	**-2.384**	-1.786	-0.963
	Ren159M20	23,456266–23,456464	0.5	0.0	**-19.789**	**-11.82**	**-6.37**	**-4.141**	**-2.915**	**-2.1**	-1.061
**CFA20**	***MITF***	24,701418–24,735483									
	Ren100j13	25,668407–25,668570	0.5	0.0	**-24.585**	**-14.621**	**-7.791**	**-4.988**	**-3.442**	**-2.417**	-1.131
	Ren178E07	26,911251–26,911383	0.5	0.0	**-12.593**	**-7.605**	**-4.164**	**-2.729**	-1.923	-1.377	-0.671

	Ren105M20	19,606338–19,606486	0.4	0.087	**-8.992**	**-5.016**	**-2.327**	-1.264	-0.709	-0.367	-0.011
	Ren67C18	22,361131–22,361265	0.5	0.0	**-11.992**	**-7.012**	**-3.606**	**-2.218**	-1.462	-0.969	-0.379
	Pax3.2*	30,185679–30,186031	0.27	0.147	**-9.89**	**-4.957**	-1.763	-0.644	-0.159	0.063	0.137
**CFA37**	***PAX3***	31,332182–31,429995									
	Pax3.1*	31,368786–31,369020	0.5	0.0	**-20.085**	**-11.159**	**-5.204**	**-2.924**	-1.779	-1.101	-0.418
	Ren75L05	31,649572–31,649607	0.5	0.0	**-9.294**	**-5.321**	**-2.65**	-1.605	-1.065	-0.732	-0.36

**Table 2:** Linkage data between the *merle *phenotype and CFA10 markers. List of the CFA10 genotyped markers in the sub-pedigree (left), the complete pedigree (middle) and all the studied pedigrees (right), with their two point Lod scores and recombination fractions (θ). All markers were selected from CanFam 1.0 sequence draft except FH2293 and C10769 taken from Guyon et al.[13]. The markers are ordered in the table from the centromere to the telomere. ^a ^markers flanking the *SOX10 *gene. Values in bold correspond to the highest Lod scores obtained in the three conditions.

			Two point Lod scores values								
			
			θ at Lod max		Lod scores at θ						
	
	*Gene*/Marker names	Marker Position (in bp)	θ	Lod max	0.001	0.01	0.05	0.1	0.15	0.2	0.3
**Lod scores in the sub-pedigree**	CFA10.1	3,109459–3,109703	0.00	6.32	6.31	6.22	5.79	5.23	4.64	4.02	2.68
	CFA10.2	3,150322–3,150695	0.00	6.92	6.91	6.82	6.37	5.78	5.16	4.50	3.05
	CFA10.3	3,169429–3,169640	0.00	3.01	3.01	2.95	2.72	2.42	2.10	1.76	1.06
	CFA10.4	3,452451–3,452757	0.04	5.94	4.82	5.71	5.90	5.55	5.04	4.44	3.04
	CFA10.5 ^c^	3,533969–3,534312	0.00	6.92	6.91	6.81	6.35	5.73	5.09	4.40	2.90
	CFA10.6	4,050670–4,050915	0.00	3.01	3.01	2.96	2.74	2.46	2.17	1.86	1.20
	CFA10.7	4,901919–4,902276	0.00	**7.23**	7.21	7.11	6.65	6.04	5.39	4.71	3.20
	CFA10.8	6,938580–6,938928	0.00	**7.23**	7.21	7.11	6.65	6.04	5.39	4.71	3.20
	CFA10.9	8,539383–8,539710	0.50	0.00	-20.08	-11.15	-5.16	-2.85	-1.68	-0.98	-0.31
	CFA10.10	9,459108–9,459324	0.07	2.62	1.21	2.15	2.60	2.57	2.40	2.16	1.52
	CFA10.11	10,599594–10,599989	0.04	5.08	3.91	4.82	5.07	4.79	4.35	3.84	2.64
	CFA10.12	12,963916–12,964094	0.15	2.55	-4.49	-0.58	1.79	2.43	2.55	2.44	1.83
	CFA10.13	13,290922–13,291305	0.18	0.75	-2.99	-1.03	0.21	0.60	0.73	0.74	0.57
	CFA10.14	13,687824–13,688059	0.11	3.44	-1.49	1.42	3.09	3.43	3.36	3.10	2.25
	CFA10.15	15,287513–15,287805	0.14	2.22	-2.99	-0.06	1.69	2.15	2.21	2.08	1.51
	CFA10.16	16,325750–16,325992	0.09	3.67	0.61	2.53	3.55	3.66	3.49	3.19	2.33
	CFA10.17	16,883188–16,883575	0.12	2.95	-2.09	0.83	2.53	2.92	2.91	2.72	2.02
	CFA10.18	17,574178–17,574420	0.08	3.87	0.91	2.82	3.79	3.84	3.61	3.25	2.31
	CFA10.19	18,220318–18,220600	0.10	4.20	-0.58	2.31	3.93	4.20	4.07	3.74	2.75
	CFA10.20	19,075239–19,075433	0.12	3.19	-1.79	1.12	2.81	3.17	3.13	2.90	2.10
	CFA10.21	19,999011–19,999231	0.26	0.51	-11.69	-5.76	-1.88	-0.50	0.12	0.41	0.47
	CFA10.22	21,607022–21,607316	0.10	4.45	-0.28	2.61	4.20	4.45	4.30	3.95	2.90
	CFA10.23	21,956041–21,956337	0.10	4.45	-0.28	2.61	4.20	4.45	4.30	3.95	2.90
	CFA10.24	23,113457–23,113656	0.25	0.92	-13.18	-6.27	-1.77	-0.17	0.52	0.83	0.85
	CFA10.25	24,031569–24,031859	0.10	3.51	-1.19	1.70	3.28	3.51	3.34	2.97	1.93
	CFA10.26	25,504535–25,504764	0.14	2.73	-4.19	-0.29	2.05	2.65	2.73	2.57	1.89
	CFA10.27	25,930447–25,930692	0.12	1.84	-1.49	0.45	1.58	1.83	1.81	1.67	1.16
	CFA10.28	26,491052–26,491288	0.13	1.66	-1.79	0.16	1.32	1.62	1.65	1.56	1.16
	CFA10.29	27,391154–27,391817	0.18	2.36	-6.88	-1.98	1.09	2.04	2.33	2.33	1.87
	CFA10.30	28,230039–28,230419	0.30	0.69	-18.28	-9.37	-3.47	-1.27	-0.23	0.33	0.69
	CFA10.31^a^	29,232270–29,232511	0.13	3.51	-3.28	0.61	2.90	3.46	3.49	3.27	2.45
	CFA10.32^a^	29,355913–29,355989	0.14	3.09	-3.88	0.02	2.37	2.99	3.08	2.94	2.24
	CFA10.33^a^	29,483066–29,483109	0.13	2.40	-2.69	0.23	1.95	2.37	2.38	2.22	1.58
	CFA10.34^a^	29,826073–29,826290	0.14	2.52	-2.69	0.24	2.00	2.45	2.51	2.38	1.81
	CFA10.35^a^	29,834441–29,834690	0.16	2.74	-6.28	-1.39	1.63	2.51	2.73	2.67	2.08
	***SOX10***	29,856920–29,867728									
	CFA10.36^a^	29,861299–29,861547	0.08	3.60	0.61	2.52	3.51	3.58	3.37	3.02	2.10
	CFA10.37^a^	29,868038–29,868267	0.14	3.38	-3.58	0.32	2.67	3.29	3.37	3.21	2.47
	CFA10.38^a^	30,176995–30,177378	0.11	3.65	-1.19	1.71	3.35	3.65	3.55	3.26	2.38
	FH2293^a^	31,696028–31,696274	0.13	3.56	-3.28	0.61	2.93	3.50	3.54	3.34	2.53
	C10.769^a^	36,663349–36,663563	0.18	1.50	-5.99	-2.06	0.42	1.20	1.46	1.49	1.18

**Lod scores in the complete pedigree**	CFA10.1	3,109459–3,109703	0.00	11.74	11.72	11.54	10.70	9.61	8.46	7.27	4.71
	CFA10.2	3,150322–3,150695	0.02	11.97	11.13	11.91	11.63	10.64	9.48	8.20	5.38
	CFA10.3	3,169429–3,169640	0.00	6.62	6.61	6.50	5.98	5.30	4.60	3.86	2.33
	CFA10.4	3,452451–3,452757	0.03	13.82	11.73	13.46	13.65	12.67	11.36	9.88	6.55
	CFA10.5 ^c^	3,533969–3,534312	0.02	8.77	7.82	8.65	8.58	7.88	7.01	6.03	3.86
	CFA10.6	4,050670–4,050915	0.00	6.92	6.91	6.79	6.24	5.51	4.76	3.99	2.41
	CFA10.7	4,901919–4,902276	0.00	**15.65**	15.63	15.39	14.30	12.88	11.40	9.84	6.54
	CFA10.8	6,938580–6,938928	0.02	**14.90**	14.13	14.87	14.39	13.16	11.72	10.17	6.76
	CFA10.9	8,539383–8,539710	0.19	2.48	-14.38	-5.54	0.02	1.78	2.38	2.47	1.89

**Lod scores in the complete pedigree plus families** ** of dachshund, Beauce shepherd and Border collie**	CFA10.1	3,109459–3,109703	0.04	10.67	8.43	10.21	10.61	9.91	8.91	7.74	5.09
	CFA10.2	3,150322–3,150695	0.04	10.97	8.73	10.51	10.91	10.21	9.21	8.04	5.39
	CFA10.3	3,169429–3,169640	0.00	6.02	6.01	5.91	5.44	4.84	4.22	3.56	2.20
	CFA10.4	3,452451–3,452757	0.03	14.38	12.33	14.05	14.19	13.15	11.79	10.25	6.83
	CFA10.5 ^c^	3,533969–3,534312	0.02	8.77	7.82	8.65	8.58	7.89	7.02	6.05	3.90
	CFA10.6	4,050670–4,050915	0.03	6.99	6.01	6.86	6.85	6.23	5.46	4.61	2.82
	CFA10.7	4,901919–4,902276	0.00	**19.87**	19.83	19.52	18.11	16.28	14.37	12.37	8.15
	CFA10.8	6,938580–6,938928	0.00	**19.57**	19.53	19.22	17.82	15.99	14.08	12.09	7.91
	CFA10.9	8,539383–8,539710	0.16	4.23	-11.97	-3.16	2.25	3.83	4.22	4.10	3.02
