# Supplementary material for: Coat colour in dogs: identification of the Merle locus in the Australian shepherd breed
Source: BMC Vet Res. 2006 Feb 27;2:9. doi: 10.1186/1746-6148-2-9 (PMC1431520; doi:10.1186/1746-6148-2-9)
Supplement: Additional File 1 — Characteristics of the markers used in the genetic linkage studies. a CFA : Canis familiaris chromosome.b Starred markers were selected from the CanFam 1.0 canine sequence draft, c marker corresponding to marker FH2537 from Guyon et al. [13], d number of alleles as determined from the sub-pedigree. E Markers flanking SOX10 gene. [file 1746-6148-2-9-S1.doc]

| CFA^a^ | Gene/ Markers^b^ | Gene/Marker Position in bp | Forward Primer | Reverse Primer | Size (in bp) | Motif | Number of Allele ^d^ |
| --- | --- | --- | --- | --- | --- | --- | --- |
| CFA11 | ***TYRP1*** | 34,664779-34,683148 |  |  |  |  |  |
|  | FH2319 | 35,863893-35,864169 | GGAACACTGTATCCTCAGTGTCC | TGGGAAGGAAGGAAGTGTTG | 255 | (AAGA)_47_ | 13 |
|  | Ren105l03 | 39,857761-39,857999 | GGAATCAAAAGCTGGCTCTCT | GAGATTGCTGCCCTTTTTACC | 239 | (TG)_25.5_ | 5 |
|  | CFA10.1* | 3,109459-3,109703 | TGGTCAATGCCTGATGAAAA | AGGAGCACAGCCAGACAACT | 245 | (AC)26 | 3 |
|  | CFA10.2* | 3,150322-3,150695 | GGGTATTAGGTTACAAGCCACTG | CCCACATTCCCCTTTCTTTA | 374 | (GATA)36 | 4 |
|  | CFA10.3* | 3,169429-3,169640 | CTGGAAGTCCATTTCCCTGA | AACAGGCGGTTCTTTCAAAC | 212 | (AC)16 | 2 |
|  | CFA10.4* | 3,452451-3,452757 | GAAATAAACCTGAAGGATTAGGC | TGTAGACAGTTTGCGGTGCT | 307 | (TTTC)20.2+(TCCT)14.2 | 8 |
|  | CFA10.5* ^c^ | 3,533969-3,534312 | TCAACCTCATTGAGACCCAAG | TTGCGGTCTAGGCCAAATAG | 344 | (TTTC)24.8 | 5 |
|  | CFA10.6* | 4,050670-4,050915 | AGCCCTTTGTAGTGGGAGTG | CCTTGTTTAACTGCCTTTGGA | 246 | (TTCT)28.2 | 3 |
|  | CFA10.7* | 4,901919-4,902276 | ATCTCTGGTTCAGGCCACTG | AGAGCAGTGAGGGGATTcct | 358 | (TTTC)36.5 | 8 |
|  | CFA10.8* | 6,938580-6,938928 | CCCTTTACCCACTTTGCTCA | AAAACCCAGAAAATTCAAAGAGA | 349 | (TTTC)64.8 | 5 |
|  | CFA10.9* | 8,539383-8,539710 | AGAAATATGTATTTGGGGCACCT | GGTCAGCCTATAGTTTCAACACAA | 328 | (AGAA)24 | 6 |
|  | CFA10.10* | 9,459108-9,459324 | GCCTCATTCTCACAAAGCTG | GCTGGACAAGTCTTGGGAAC | 217 | (AC)30.5 | 3 |
|  | CFA10.11* | 10,599594-10,599989 | GCCAAGCTGAACTAGCCTGT | GGAAATCTTAGAGAACATAATTGTGAA | 396 | (AAAG)41.5 | 5 |
|  | CFA10.12* | 12,963916-12,964094 | CCATGACTCCCTCTCTCTGG | CATGGCTGATGGATCCAAGT | 179 | (TTTC)22 | 6 |
|  | CFA10.13* | 13,290922-13,291305 | AATCACGCAAGATTGCACAC | ACCGTTAGACTCACCGAACG | 384 | (TAGA)62 | 3 |
|  | CFA10.14* | 13,687824-13,688059 | TGGAACCAGTAACTTAGAAAGCA | TTTAGCAAGTGAAAGCAACCAA | 236 | (AGGA)31.2 | 7 |
|  | CFA10.15* | 15,287513-15,287805 | CTGGGAATAAAAATTAGCTTGC | GCCTGCCAGTCAACAGAGG | 293 | (TTTC)41 | 5 |
|  | CFA10.16* | 16,325750-16,325992 | GGAGAAAACCTTGGCAATGA | GTGAAGTCCCCAAAGCAAAA | 243 | (TG)22.5 | 6 |
|  | CFA10.17* | 16,883188-16,883575 | AACGTCAACACCACCAGACA | TTTCTTGAGTAACGAGATTTCCA | 388 | (ATCT)68 | 8 |
|  | CFA10.18* | 17,574178-17,574420 | ACATCCTATGTATGTCTAATGAAGGA | ACTCCATTTGGGGCCTTTT | 243 | (AAAG)40.5 | 6 |
|  | CFA10.19* | 18,220318-18,220600 | AGATGGCAATTGGCAAAGAC | TGAATGACGACTGTGAATTTGA | 283 | (TTCT)28.8 | 5 |
|  | CFA10.20* | 19,075239-19,075433 | GACCTACGCCCTCAGCAATA | TGCTGGCAGATATCTTTGCTT | 195 | (TG)31.5 | 4 |
|  | CFA10.21* | 19,999011-19,999231 | TCTGAAGGCACCAGTGTCAG | TAGTCACCCCAGGAGACAGG | 221 | (GT)28.5 | 3 |
|  | CFA10.22* | 21,607022-21,607316 | CCTGTCTGCTCCACCATTTT | CCACACTTAGCAGGGAGTCG | 295 | (TTCT)36 | 7 |
|  | CFA10.23* | 21,956041-21,956337 | TGCTTGAGAGGTTTGTTTGC | GATTCAACTTTGACATTTGATCTTATT | 297 | (AAAG)40.8 | 7 |
|  | CFA10.24* | 23,113457-23,113656 | TGTGAGCAGAGTGTGCAAAA | GTGCGGTAAAGCTCGTGAAT | 200 | (GAAG)20.2 | 5 |
|  | CFA10.25* | 24,031569-24,031859 | CCCTAACAAATTTCTAGTTCTTGC | CACCTGAAACTAATATAACCCTGTATG | 291 | (TTTC)44.8 | 7 |
|  | CFA10.26* | 25,504535-25,504764 | TTGCAGAGCTACAGGTGGTG | ACTGAGCGAATGAGCCAACT | 230 | (GT)24.5 | 5 |
|  | CFA10.27* | 25,930447-25,930692 | AGAAGCATCCCTGCTTTCAA | GAGCCTGCCTGAAAATGAAG | 246 | (TG)20.5 | 2 |
|  | CFA10.28* | 26,491052-26,491288 | CACGTCAGGCTCTCTGCAT | CAGAGCTGCCTTGCAATAAA | 237 | (TG)19.5 | 4 |
|  | CFA10.29* | 27,391154-27,391817 | ACCTCTCCCTCTGCTTGTGA | CAGGACAGGCACAGTGAATC | 355 | (AAAG)65.2 | 6 |
|  | CFA10.30* | 28,230039-28,230419 | TGACTGGCTTACCAACATGAA | AGCTCCACATCAGGCTCAGT | 381 | (TATT)51.5 | 8 |
|  | CFA10.31* ^e^ | 29,232270-29,232511 | GCACCACGTTGAGACTCCTT | GGTCAAGGTGAGGAGACCAG | 242 | (TTTC)28.2 | 8 |
|  | CFA10.32* ^e^ | 29,355913-29,355989 | TTCCTCTGTTACCTCAGTTCTGC | CTGCTTCTCCCTCTCCCTCT | 194 | (TCTT)19.5 | 6 |
|  | CFA10.33* ^e^ | 29,483066-29,483109 | TAGGGCATGATCCTGGAGAC | AGGCTGGGGGATGTAGTTTT | 227 | (GT)22 | 5 |
|  | CFA10.34* ^e^ | 29,826073-29,826290 | TCAATCTGCCTGGTCCTACC | GGGGAAGGGACCTTGAACTA | 218 | (AC)21 | 4 |
|  | CFA10.35* ^e^ | 29,834441-29,834690 | CCCCACCCTCTTTCTCAAAT | TTTCATGGGTTGTGCATTTA | 250 | (GAAA)34.2 | 4 |
| **CFA10** | *SOX10* | 29,856920-29,867728 |  |  |  |  |  |
|  | CFA10.36* ^e^ | 29,861299-29,861547 | GGCTCTCTGGCAGCTCTCT | CAACGAAGGTCTCTCCAAGG | 249 | (AC)25 | 7 |
|  | CFA10.37 ^*e^ | 29,868038-29,868267 | CATTTGCCCAACAGAGAGGT | CTTGGCTAGATCCTGGAGCA | 230 | (AC)25.2 | 6 |
|  | CFA10.38* ^e^ | 30,176995-30,177378 | CAACCCTTCCAAGCTCCAT | GCCGCTGAAAGAACAATCTAA | 384 | (TTCC)18.5+(TCTT)46.5 | 11 |
|  | FH2293 ^e^ | 31,696028-31,696274 | GAATGCCCTTCACCTTGAAA | AGGAAAAGGAGAGATGATGCC | 247 | (GAAA)49.8 | 8 |
|  | C10.769 ^e^ | 36,663349-36,663563 | CCATTCATGTTGTTGCAGATG | TGCTCCCCTGTGTTCTGAG | 215 | (GT)18 | 4 |
|  | Ren96j16 | 22,981531-22,981669 | CCATGACTCAAGGGAACT | TGAAGGAGAAAGGCAGAGA | 139 | (TG)_15.5_ | 5 |
|  | Ren130E03 | 22,981511-22,981683 | AAGACAGAAGCCCAAGATA | GAAGAGGCTGAGAGTGAAG | 173 | (TG)_15.5_ | 6 |
|  | Ren159M20 | 23,456266-23,456464 | ATGGCCAGAAAGCAAGAAGA | TGGCCTGAAGATTGTAAATGA | 199 | (AC)_18.5_ | 4 |
| CFA20 | MITF | 24,701418-24,735483 |  |  |  |  |  |
|  | Ren100j13 | 25,668407-25,668570 | TGATTGACTCTACTTTACACA | TATATTAGGCGGTTTTCTTCT | 164 | (GT)_15.2_ | 3 |
|  | Ren178E07 | 26,911251-26,911383 | TTGCAAATGGCAAAATTTCA | GGATACAACTCAAGTACCCATCC | 133 | (AC)_12.5_ | 2 |
|  | Ren105M20 | 19,606338-19,606486 | ACCCCTGATAACAACTTAGT | TAAAACAACAATTCATCTGTC | 149 | (CA)_17_ | 4 |
|  | Ren67C18 | 22,361131-22,361265 | TCTGTGCGTTTCCGTTTATG | TTAGTACCTGTTTGTTATCC | 135 | (CA)_20_ | 5 |
|  | Pax3.2* | 30,185679-30,186031 | ATAATCAGGCCAGCCCTTCT | ACAATACAGCTGGCCACAGA | 353 | (TTCC)_13.8_+ (TTCT)_44.2_ | 9 |
| CFA37 | PAX3 | 31,332182-31,429995 |  |  |  |  |  |
|  | Pax3.1* | 31,368786-31,369020 | CCAGGGATGCTAGGATTTGA | TCGGAATTCCTGTTTGGATT | 242 | (CT)_26.5_+ (CA)_23_ | 4 |
|  | Ren75L05 | 31,649572-31,649607 | AGTAAATGAGCAAGTAGACA | TCATACGTGTACCAGAGTT | 106 | (CA)_18_ | 4 |
