# Supplementary figures and images for: Coat colour in dogs: identification of the Merle locus in the Australian shepherd breed
Source: BMC Vet Res. 2006 Feb 27;2:9. doi: 10.1186/1746-6148-2-9 (PMC1431520; doi:10.1186/1746-6148-2-9)

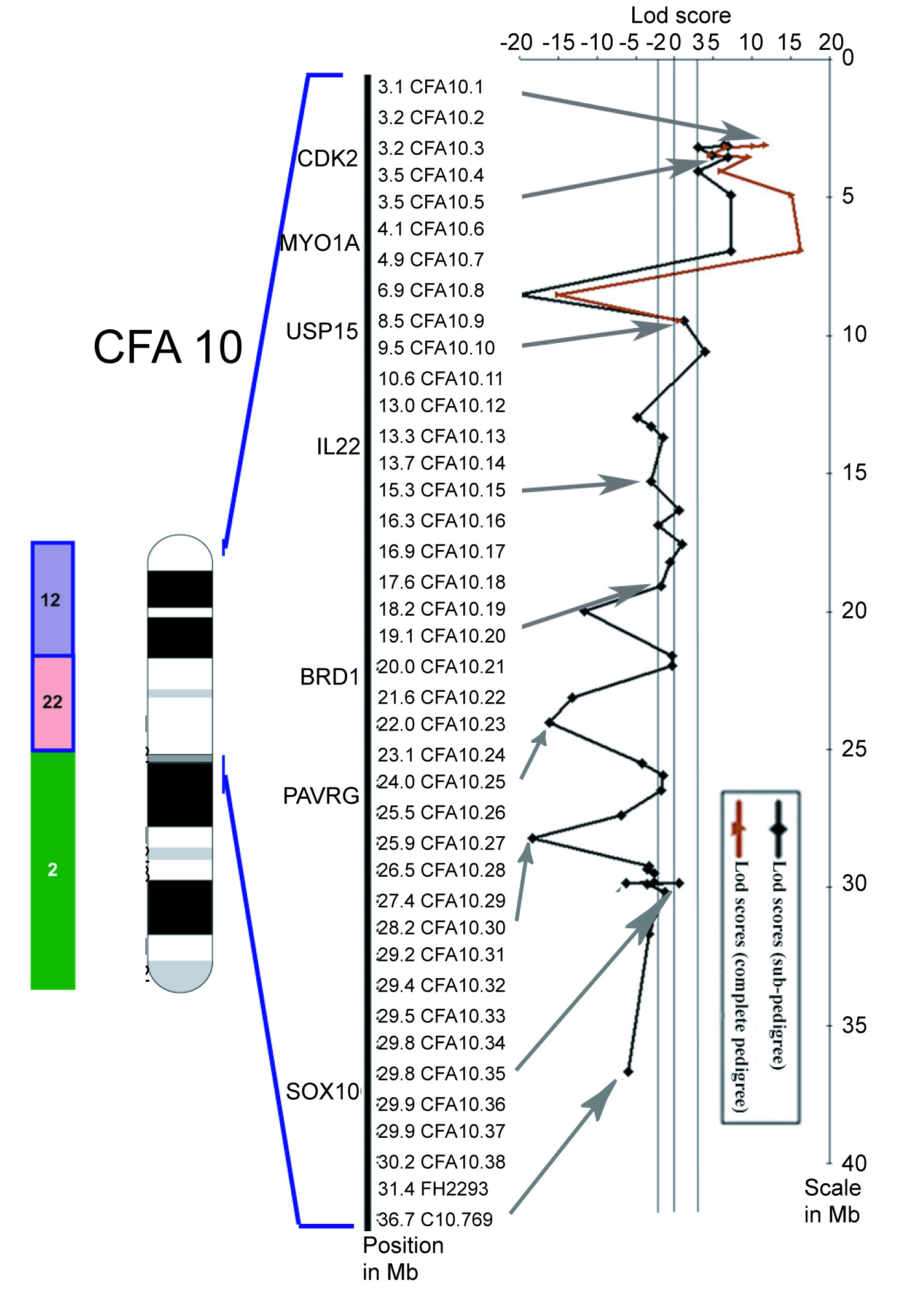

Supplement: Additional File 2 — Scheme of two-point linkage analysis of the merle phenotype in the Australian Shepherd dog pedigrees on CFA10. Two-point linkage analysis of the merle phenotype in the Australian shepherd dog sub-pedigree (in black) and complete pedigree (in brown) (Lod scores at theta = 0) is shown on the right. An ordered list of genotyped markers (right) and genes (left) and their position in Mb are indicated in the middle. An ideogram of the canine chromosome 10 is shown on the left with the corresponding human chromosomal conserved segments. NB: genomic sequence systematically starts at an arbitrary coordinate of 3 Mb to include the non-sequenced centromeric region. [file 1746-6148-2-9-S2.jpeg]
